# Supplementary material for: Ultrasound-guided dynamic needle tip positioning technique for radial artery cannulation in elderly patients: A prospective randomized controlled study
Source: PLoS One. 2021 May 14;16(5):e0251712. doi: 10.1371/journal.pone.0251712 (PMC8121362; doi:10.1371/journal.pone.0251712)
Supplement: S1 Table — (DOCX) [file pone.0251712.s002.docx]

**S1 Table. Logistic regression analysis of factors related to first-attempt success using the backward likelihood ratio method**

|  | Univariate analysis | | | Multivariate analysis* | | |
| --- | --- | --- | --- | --- | --- | --- |
|  | OR | 95% CI | *P* value | OR | 95% CI | *P* value |
| Use of ultrasound-guided DNTP technique | 4.33 | 2.29-8.16 | < 0.001 | 4.33 | 2.34-8.00 | < 0.001 |
| Age | 1.02 | 0.96-1.08 | 0.54 |  |  |  |
| Sex | 1.10 | 0.52-2.33 | 0.81 |  |  |  |
| ASA |  |  |  |  |  |  |
| 1 | ref. |  |  |  |  |  |
| 2 | 0.56 | 0.13-2.36 | 0.43 |  |  |  |
| 3 | 0.57 | 0.12-2.77 | 0.48 |  |  |  |
| BMI | 1.03 | 0.94-1.13 | 0.54 |  |  |  |
| Hypertension | 0.75 | 0.36-1.57 | 0.44 |  |  |  |
| Diabetes mellitus | 1.41 | 0.69-2.89 | 0.34 |  |  |  |
| Hypercholesterolemia | 1.19 | 0.60-2.34 | 0.62 |  |  |  |
| Peripheral vascular disease | 1.57 | 0.65-3.75 | 0.32 |  |  |  |
| History of smoking | 0.82 | 0.26-2.60 | 0.73 |  |  |  |
| Diameter of the radial artery | 2.41 | 1.12-5.21 | 0.03 | 2.15 | 1.08-4.30 | 0.03 |
| Depth of the radial artery | 1.08 | 0.77-1.50 | 0.67 |  |  |  |
| SBPstart | 1.00 | 0.98-1.01 | 0.55 |  |  |  |
| HRstart | 1.01 | 0.99-1.03 | 0.52 |  |  |  |

*Hosmer and Lemeshow test, p-value = 0.561; Nagelkerke R^2^ = 0.158
OR, odds ratio; CI, confidence interval; DNTP, dynamic needle tip positioning; ASA, American Society of Anaesthesiologist physical status classification; ref., reference; BMI, body mass index; SBPstart, systolic blood pressure at the start of the procedure; HRstart, heart rate at the start of the procedure.
