# Supplementary material for: Ultrasound-guided dynamic needle tip positioning technique for radial artery cannulation in elderly patients: A prospective randomized controlled study
Source: PLoS One. 2021 May 14;16(5):e0251712. doi: 10.1371/journal.pone.0251712 (PMC8121362; doi:10.1371/journal.pone.0251712)
Supplement: S1 Text — (PDF) [file pone.0251712.s003.pdf]

Institutional Review Board

Hanyang University Hospital

222-1 Wangsimni-ro, Seongdong-gu, Seoul 133-792 KOREA
Phone: 82-2-2290-9653 / FAX: 82-2-2290-9272
Chairman: Prof. Shin, In Chul, MD. Ph.D. (icshin@hanyang.ac.kr)

Investigator
Name: Kyu Nam Kim
Institute for the Research Department of Anesthesiology and Pain Medicine
Hanyang University College of Medicine Seoul 133-792, Korea

Approval of the study (Ultrasound-guided Dynamic Needle Tip Positioning Technique versus Palpation Technique for Radial Artery Cannulation in Elderly Patients **) from the Institutional Review Board on Human Subjects Research and Ethics**

Committee, Hanyang University Hospital, Seoul, Korea
Approval Date : 2018. 12. 09

Dear Investigator

The above-mentioned study was presented for examination to the members of Institutional Review Board (IRB) of our Hospital. No ethical or legal concerns were raised against carrying out the study. The IRB can therefore grant the study the seal of ethical and legal admissibility.

As a precautionary measure, I would like to point out that, even with a positive appraisal of the project by the IRB, the absolute medical and legal responsibility for implementation of the project remains entirely with you and your staff.

You are requested to inform the IRB of any serious or unexpected incidents which may occur in connection with this study. Any amendments to the study protocol will also have to be reported to the IRB.

I wish you every success with your study.

Sincerely

Shin, In Chul, **MD. Ph.D.**
Chairman
Institutional Review Board on Human Subjects Research and Ethics Committee
Hanyang University Hospital, Seoul, Korea
E-mail: (icshin@hanyang.ac.kr)

Institutional Review Board

Hanyang University Hospital

222-1 Wangsimni-ro, Seongdong-gu, Seoul 133-792 KOREA
Phone: 82-2-2290-9653 / FAX: 82-2-2290-9272
Chairman: Prof. Shin, In Chul, MD. Ph.D. (icshin@hanyang.ac.kr)

Approval for the study from the Institutional Review Board on Human Subjects Research and Ethics Committee
Hanyang University Hospital, Seoul, Korea

**Approval No**	**IRB File No. HYUH 2018-10-024-001**
**Approval Date**	**2018. 12. 09.**
**Title**	Ultrasound-guided Dynamic Needle Tip Positioning Technique versus Palpation Technique for Radial Artery Cannulation in Elderly Patients
Directors of the research	Kyu Nam Kim
Institute for the Research	Department of Anesthesiology and Pain Medicine<br>Hanyang University College of Medicine
Decision from the Committee	Approve the study as proposed.

After reviewed thoroughly by the Institutional Review Board on Human Subjects Research and Ethics Committee, Hanyang University Hospital, Seoul, Korea, this letter certificate that the proposed research is approved.

2019. 11. 21.

Institutional Review Board
on Human Subjects Research and Ethics Committee

Committee Chair: Professor Shin, In Chul , MD. Ph.D.
