# Supplementary material for: Ultrasound-guided dynamic needle tip positioning technique for radial artery cannulation in elderly patients: A prospective randomized controlled study
Source: PLoS One. 2021 May 14;16(5):e0251712. doi: 10.1371/journal.pone.0251712 (PMC8121362; doi:10.1371/journal.pone.0251712)
Supplement: S1 File — (DOC) [file pone.0251712.s004.doc]

**1. Study Title**

Ultrasound-guided Dynamic Needle Tip Positioning Technique versus Palpation Technique for Radial Artery Cannulation in Elderly Patients

**2. Study Site**

Hanyang University Seoul Hospital

Address: 222-1, Wangsimni-ro, Seongdong-gu, Seoul, Korea (04763)

**3. Research director (Investigators) and department**

Kyu Nam Kim, Department of Anesthesiology and Pain Medicine Hanyang University Seoul Hospital

Soo Yeon Kim, Department of Anesthesiology and Pain Medicine, Hanyang University Seoul Hospital

**4. Sponsors**

There is no sponsor.

**5. Purpose of study**

Arterial cannulation is a procedure often performed for repetitive blood tests and real-time monitoring of patient blood pressure during surgery.(1) The arterial line can be placed at various locations, but the radial artery is the most commonly used blood vessel due to its easy accessibility and presence of dual supply to the hands through the ulnar artery.(2) However, arterial cannulation is often difficult in the hard-to-catheterize radial artery in elderly patients because they often have age-related arterial wall changes and tortuous arteries due to various underlying diseases.(3-5)

Recently, ultrasound with a dynamic needle tip positioning (DNTP) technique was introduced.(6) With DNTP, the probe moves along the arteries in small increments, and the needle is advanced in the same direction. None of studies investigated the ultrasound-guided DNTP technique in elderly patients. We hypothesized that the DNTP technique would have a higher success rate and reduce the incidence of complications.

**6. Detailed Description of Study Protocol**

**1) Randomization and allocation concealment**

Patients enrolled in the study were allocated to either the ultrasound-guided DNTP technique group (DNTP group) or the palpation method group (palpation group) with a 1:1 ratio. Randomization was performed by an independent person using a computer-generated random number list. The allocation results were sealed in envelopes that were opened just before artery cannulation.

**2) Blinding**

It was not possible to blind cannulation practitioners to method used. However, enrolled participants were blinded, and a separate observer who was blinded to patient group measured the diameter and depth of the radial artery and recorded the outcomes. A barrier was placed between the practitioner and the outcome observer.

**3) Interventions**

This study is aimed at patients undergoing general anesthesia who have fully understood and agreed to this study. The cannulation practitioner selects the right or left arm for radial artery cannulation depending on surgery site, location of blood pressure cuff, and his/her preference. At the level of the radial styloid process, the diameter and depth of the radial artery are measured using ultrasound equipment. After disinfection of the cannulation site, the radial artery cannulation is performed using either the ultrasound-guided DNTP technique or the palpation method. We plan to check the success or failure of the procedure, cannulation time, the number of attempts and redirections, blood pressure and heart rate before and after cannulation and incidence of complications related to the procedure.

**7. Expected Duration of Study**

About 19 months: from when the ethics committee approve the trial (approximately December 2018) to 30 June 2020 including data acquisition, analysis and manuscript period.

**8. Eligibility Criteria and Estimated Enrollment**

**1) Inclusion criteria:**

- Patients undergoing general anesthesia

- American Society of Anesthesiologist(ASA) physical status classification I, II, III

- Age 65 years or older

- If arterial cannulation is required for surgery

**2) Exclusion criteria:**

- Hemodynamically unstable patients (systolic blood pressure 60 or less)

- If arterial cannulation cannot be performed at radial artery

- Skin abnormality, inflammation sign, hematoma at radial arterial cannulation site

- Abnormal tesult test for Modified Allen test

- History of hand or wrist surgery

**3) Sample size calculation:**

The number of samples was calculated by referring to a randomized controlled trial comparing ultrasound-guided DNTP and the palpation method for radial artery cannulation. The first-attempt success rates were 83% and 48%, respectively. Therefore, we expected that the first-attempt success rate would be 50% using the palpation method in our study. We assumed that it would be meaningful when the success rate increased by 20% with the ultrasound-guided DNTP technique. Estimating the number of samples at significance level (α) 0.05 and power 90%, a total of 253 subjects was required. Therefore, 256 subjects (128 in each group) were enrolled to account for a 1% dropout rate (PASS 14 Power Analysis and Sample Size Software, 2015. NCSS, LLC. Kaysville, UT, USA).

**9. Outcome measures and Interpretation of Results (Statistical analysis)**

**1) Outcome measures**

The primary outcome is first-attempt success rate. Success is confirmed when an arterial waveform is observed on the vital signs monitor. The secondary outcomes are overall success rate, numbers of attempts and redirections, cannulation time, and incidence of complications (hematoma, thrombosis, spasm, and ischemia).

**2) Statistical analysis**

Statistical analysis is performed with SPSS software version 24. Categorical data are expressed as number of patients (percentage) and compared using Pearson’s χ2 test or Fisher’s exact test. Continuous data are compared using Student’s t-test or the Mann-Whitney U-test and. A normality test for continuous data was performed using the Shapiro-Wilk test. P values < 0.05 were considered statistically significant.

**10. Ethical considerations for research**

**Method to secure research ethics**

1) This study must be conducted in compliance with the standards of the Helsinki Declaration and all applicable regulations of International Conference on Harmonization-Good Clinic Practice (ICH-GCP)

2) This study was performed after approval by the Hanyang University Hospital Institutional Review Board

3) The purpose and procedures of the study were explained to eligible patients, and written informed consent was obtained.

4) Participants can quit the study at any time during the study by voluntary will.

5) All data of the subject of this study is anonymized, strictly confidential, and a password is set and stored as a separate file.

**11. References**

1. Scheer BV, Perel A, Pfeiffer UJ. Clinical review: Complications and risk factors of peripheral arterial catheters used for haemodynamic monitoring in anaesthesia and intensive care medicine. Critical Care. 2002;6(3):199-204.

2. Brzezinski M, Luisetti T, London MJ. Radial Artery Cannulation: A Comprehensive Review of Recent Anatomic and Physiologic Investigations. Anesthesia & Analgesia. 2009;109(6):1763-81.

3. Xu X, Wang B, Ren C, Hu J, Greenberg DA, Chen T, et al. Age-related impairment of vascular structure and functions. Aging and Disease. 2017;8(5):590-610.

4. Thijssen DHJ, Carter SE, Green DJ. Arterial structure and function in vascular ageing: Are you as old as your arteries? Journal of Physiology. 2016;594(8):2275-84.

5. Han H-C. Twisted Blood Vessels: Symptoms, Etiology and Biomechanical Mechanisms. Journal of Vascular Research. 2012;49(3):185-97.

6. Clemmesen L, Knudsen L, Sloth E, Bendtsen T. Dynamic Needle Tip Positioning – Ultrasound Guidance for Peripheral Vascular Access. A Randomized, Controlled and Blinded Study in Phantoms Performed by Ultrasound Novices. Ultraschall in der Medizin - European Journal of Ultrasound. 2012;33(07):E321-E5.
